# The Promise and the Hype of ‘Personalised Medicine’

**DOI:** 10.1080/20502877.2017.1314886

**Published:** 2017-05-18

**Authors:** Tim Maughan

**Affiliations:** ^a^ CRUK/MRC Oxford Institute for Radiation Oncology, University of Oxford, Oxford, UK

**Keywords:** personalised medicine, cancer, stratified medicine, promise, hype, precision medicine

## Abstract

Personalised medicine is widely considered as the way of the future for medicine. However, progress in cancer, with a few outstanding exceptions, has fallen below expectations because of the challenges of tumour heterogeneity and clonal evolution. In both benign and malignant disease, diseases caused by single genetic alterations are more amenable to precision medicine approaches. However, most common diseases are caused by a complex interplay of multiple genetic and environmental factors making personalised medicine far more challenging. The current optimism for personalised medicine is distorting clinical consultations, resource allocation and research funding prioritisation. A research active clinician must act both as an agent of change and development, and as a communicator of realism. Thus personalised medicine that includes a sober appreciation of what genomics can achieve, together with continued focus on the individual as a person not just as a genome, will contribute to further improvements in health and healthcare.

Personalised medicine has become a catch phrase filled with promise. Promise that you as an individual will be able to receive the right treatment for your specific medical need at the right time and at the right dose. The European Alliance for Personalised Medicine goes even further and includes the right prevention for as yet undiagnosed diseases and health risks. ‘Personalised’ carries the notion of individualised — this is exclusively designed for you. The more scientifically rigorous though less accessible term, stratified medicine, seeks to identify groups or strata of patients with specific molecular characteristics or other determining factors which predict prognosis and response to therapy. The Medical Research Council in its stratified medicine programme claims that stratified medicine will ‘ensure’ you get the right treatment at the right time. These are very significant claims. Are they realistic and truthful or do they reflect an environment in which the promise of personalised medicine has become disconnected from reality? If so why has this occurred and what are the potential risks of that disconnection?

## Personalised medicine for cancer

Personalised medicine can be defined as treatment which targets the causative molecular alteration of a disease in the individual which is specific to that individual's disease and is, as a consequence of this individual treatment selection, highly effective. The paradigm for this was established in the treatment of a relatively rare subtype of leukaemia, chronic myeloid leukaemia, CML. CML is caused by a single aberrant protein (the bcr-abl kinase) encoded by a novel oncogene (the bcr-abl fusion gene) located at the junction point of a consistent chromosomal translocation affecting chromosomes 9 and 22, resulting in the formation of an abnormal and pathogenic alteration named the Philadelphia chromosome. Novartis developed a highly specific inhibitor of the bcr-abl kinase, an agent called imatinib. In the phase 1 trial adverse events were noted to be minimal and 53 of 54 patients on the trial went into complete haematological response (Druker *et al.* 2001, p. 1031). Five years later the same author reported that 83% of those patients remained disease free, compared to a historic rate of overall survival of about 30% (Druker *et al.* 2006, p. 2408). This dramatic breakthrough has revolutionised therapy for CML, and has also revolutionised expectations, creating a new paradigm of targeted therapies directed at genetic driver lesions. The trouble is that in retrospect the results from imatinib in CML stand out as one of a very few targeted agents to deliver long lasting benefit as single agents. Other outstanding breakthrough therapeutics in the last 20 years of comparable clinical benefit can be numbered on the fingers of one hand and include rituximab (Maloney *et al*. 1994, p. 2457) and more recently ibrutinib for B cell lymphoma (Byrd *et al.* 2013, p. 32), trastuzumab for her-2 positive breast cancer (Vogel *et al*. 2002, p. 719) and most recently the immune checkpoint inhibitors targeting CTLA-4 (Hodi *et al.* 2010, p. 711) and PD-1 (Robert *et al*. 2015, p. 320). Despite these major improvements, the overall trend of limited clinical benefit was summarised by Tito Fojo who reported that the mean improvement in overall survival from 71 targeted cancer therapies approved by the FDA between 2002 and 2014 was only 2.1 months (Fojo *et al*. 2014, p. 1225).

The main scientific reason that has emerged for this relative failure of targeted therapies for cancer is the presence of profound tumour heterogeneity and clonal evolution that can be identified in most cancers. Cancer is characterised by genetic instability which drives not only the initial development of the cancer, but continues to drive the process such that significantly different clones can be identified in different parts of the primary tumour and in different metastases (Gerlinger *et al.* 2012, p. 883). This clonal variation provides the cancer with multiple options for evading eradication by targeted therapy — as Bert Vogelstein has stated: ‘Resistance to targeted therapy is a fait accompli — the time to recurrence is simply the time taken for the subclone to repopulate the tumour’ (Diaz *et al.* 2012, p. 537). Thus the beneficial effect of imatinib in CML turns out to be the outlier rather than the norm, due to the very unusual fact that CML is driven by a very specific and singular genetic alteration. In contrast to nearly all other examples, cancers are driven by a mixture of genetic abnormalities which vary between different sites of origin (breast, or prostate or bowel etc) and between individuals. In addition, external environmental factors, such as diet, smoking, sunshine and exercise also significantly influence the development of most cancers.

It is also emerging that the way the host cells respond to the genetically altered cancer cells has a very major influence on the subsequent behaviour of the cancer, affecting both prognosis and response to therapy. The disruptive technology which has altered the field dramatically in the last two years has been the advent of immunotherapy. Antibodies targeting negative regulators of the immune response such as CTLA-4 and PD-1, so called immune checkpoint inhibitors, have shown significant improvements in long term survival in a small number of cancer types characterised by very high levels of mutation burden (Rizvi *et al*. 2015, p. 124). This has been a game changer in melanoma and we await with interest the wider application of this in other cancers including lung, head and neck and bladder cancer where initial promising results have led to licensing authorisation. However, selection of patients for treatment using biomarkers remains essential for immunotherapy as for targeted therapy. This was vividly revealed recently by the failure of the trial of Bristol Myers Squibb PD-1 inhibitor nivolumab in non-small cell lung cancer, compared to the success of the competitor agent, pembrolizumab from Merck: the failure was attributed to BMS's failure to stratify the patients appropriately with a biomarker (Reck *et al.* 2016, p. 1823).

The consequences of heterogeneity, clonal evolution and the influence of the host response are that simple genetic tests are much less accurate in predicting prognosis and treatment response than was expected based on the CML:imatinib paradigm. Similarly, targeted drug therapies may show an initial response, but this is rapidly overtaken by tumour regrowth due to emergence of tumour clones often demonstrating multiple different mechanisms of resistance. Despite this, personalised cancer medicine, now enhanced by immunotherapy, is still projected as the existing paradigm, and supported by major cancer centres across the world, by pharmaceutical and diagnostic companies alike. Researchers in the field and especially pharmaceutical companies are acutely aware of the challenges, but still clinicians, patients and their advocates pursue access to these targeted agents with enthusiasm.

## Non-malignant disease

Cancer has led the field in the introduction of personalised medicine, but how does this apply to other diseases where the problems of genetic instability and clonal evolution do not occur? Personalised medicine is making a contribution to both rare and more common diseases. In rare inherited disorders caused by a single gene defect, accurate genetic diagnosis is clarifying the cause of otherwise poorly understood syndromes. In cystic fibrosis, the most common recessively inherited disorder, the disease is caused by changes in a specific gene, the Cystic Fibrosis transmembrane conductance regulator (CFTR) gene, resulting in an increase in viscosity of mucous secretions especially in the lungs and pancreas. The CFTR gene is altered by one of a thousand different specific mutations in an individual resulting in loss of function. A therapeutic breakthrough has been achieved through a targeted therapy named ivacaftor for patients affected by one of these mutations (G551D) which occurs in 4% of patients (Ledford 2012, p. 482). In Huntingdon's disease where a single mutation in a specific gene has been identified for around two decades, using the new gene-editing CRISPR technology in animal models has suggested a potential new approach to therapy (Shin *et al.* 2016, p. 4566). In infective disorders, unravelling the infectious agent's genome has led to more precise therapeutic decisions. Thus in the viral induced hepatitis C, specification of the viral genome is a key factor in selecting the right treatment for the right patient (Chopra 2017).

The challenge in non-malignant disease is for the most common non communicable diseases, which are caused by a complex interplay of multiple genetic predispositions with a major overlay of environmental influences. Diabetes, asthma, hypertension, rheumatoid arthritis, psoriasis are some of the complex benign conditions being investigated for discovery of molecular stratifiers within the MRC stratified medicine consortia. The challenge is shared by the common cancers such as colorectal cancer, where multiple genes are altered in every case (Cheng *et al.* 2015) and there are significant influences on the disease by diet, exercise, the microbiome and the tumour microenvironment which are distinct from the effects of the causative genetic changes. In these complex conditions, we certainly need large datasets to enable us to test hypotheses of causation and prediction of prognosis and prediction of treatment response. The assembly of ‘big data’ sets from routinely collected data, supplemented by genetic or genomic or imaging data is underway to address these complexities. A most remarkable example of this is the UK biobank which collects data from 500,000 individuals in a prospective data collection exercise. MRI imaging data will soon supplement clinical and laboratory data in 100,000 of these individuals. While large datasets are certainly of immense value in demonstrating strong statistical associations, it remains to be proven that machine learning approaches using big data sets will be able to find true predictive tests to drive medical decision making for personalised medicine.

## The promise and the hype

The current environment of biomedical research is suffused with optimism regarding the ability of a personalised medicine approach to be able to deliver massive improvements in clinical outcomes, built on the remarkable benefits of novel therapeutics in disorders driven by a single genetic alteration. The challenge is that most diseases are far more complex. The stories of extraordinary outcomes from a very few therapeutics have achieved almost mythical proportions. Their stories are told and retold at personalised medicine conferences, with limited presentation on the many failed attempts to reproduce the benefits in more complex diseases. The question is whether there are any consequences of this retelling of success about which we should be concerned. Is this hype constructive, in engendering appropriate expectations of further success, or destructive?

## Risks to research funding

A positive consequence of retelling success is that it does provide examples of how science has overcome life threatening disease. Building on this can provide an excellent basis for applications for drug approval, grant applications and career building. I have done it myself, so do not point the finger at others without thinking about the implications for my own research programme. The challenge to the research community is to focus on failure and the reasons for failure in order to avert over-inflated expectations driven by hype. Peer review should control this research context risk. However, if the whole community is inappropriately optimistic about a field of research, as we have experienced with targeted therapy of cancer over the last 15 years and are now experiencing with immunotherapy, there is risk of bias across the whole research ecosystem. Research funding committees need to hold themselves accountable on this issue, to keep a broad view of clinical and population priorities, and avoid the temptation of getting over excited about the apparent promise of the science. Thus in cancer, if we are really to make progress in reducing mortality our focus needs to be on primary prevention, early detection and optimising treatment at the time of first diagnosis to achieve cure. Too much of the current focus is on new treatments for patients with widespread metastatic disease looking for short term benefit at immense cost for those few for whom the personalised medicine is beneficial. Funders can also be caught up in the hype. There is a risk that grant allocation to areas of apparent success can become over generous, to the detriment of other critically important areas. So researchers in population health or mental health may look askance at the quantity of funding allocated to personalised medicine, when their concern for a wider population with immense need is deprioritised. This is even more graphically illustrated by the relative lack of research funding for the diseases associated with poverty and common in the global south. This global perspective is taken up by Sullivan in this issue.

## Risks to health care cost allocation

This broader view raises the question of whether personalised medicine may only be applicable for the rich world, and risks removing funding from the care of the less privileged. This is discussed further by Gray (this volume). The narrow focus of some research and clinical care on the individual undertaking expensive molecular characterisation in search of the few people who may benefit from a particular targeted agent increases health care costs. Benefits need to be commensurate with the cost. The contrary view is that the focus on individualisation has economic benefits as it will limit the expenditure on therapies to the limited population who are most likely to benefit, thus saving health care costs. In the UK we have established an unlikely two tier system for reimbursement decisions. The National Institute of Health and Clinical Excellence (NICE) has established a strong international reputation for stringent cost effectiveness evaluation of novel interventions. The consequence has been that many licensed agents have failed to pass the cost effectiveness hurdle established by NICE. The subsequent rationing of novel medicines led to an upsurge of public outcry from patients and some physicians. The consequence, following personal lobbying of David Cameron from the leader of the rare cancers initiative, was the establishment of a second tier of funding. The Cancer Drugs Fund (CDF) was established in 2011 to provide a route for NHS patients to obtain access to agents which had failed NICE's cost effectiveness evaluation. Following repeated iterations, the CDF is now reunited with NICE into an integrated system of appraisal. This political response to public outcry deserves some reflection. Drugs that are licensed have to pass a high bar of efficacy (that they do what they claim) and of clinical effectiveness (that they do achieve measurable clinical benefit). NICE introduced a further criterion — cost effectiveness. One can readily understand the desperate cry of the individual for access to any treatment which may be beneficial for yourself or for a loved one. Was that outcry fed by a realistic understanding of the degree of benefit or by a hyped over expectation? Much of the outcry initially was driven by delays in NICE appraisal for highly effective agents such as herceptin, rituximab and imatinib, all of which are now fully reimbursed and widely used. Now the focus is appropriately on those agents pending NICE appraisal and more questionably on the margin of cost effectiveness. The Cancer Drugs Fund currently has a budget of £340 million. This is a clear example of exaggerated expectations driving a distortion of clinical commissioning.

## Risks to clinical care

In what other ways does the hype of personalised medicine over-promise and distort priorities? In clinical practice, every oncologist has long experience of the patient attending the clinic with the cutting from a newspaper or an internet page, describing the benefits of the latest novel therapy, almost invariably reported in glowing terms of ‘breakthrough’. This is now being replaced by the patient bringing the print-out of their privately purchased tumour genetic profile. The questions arise immediately. Is a drug identified as associated with an actionable mutation (obtained from any cancer type) actually going to have any effect in this person's type of cancer? How can the physician possibly choose between the many suggested drugs listed alongside the multiple mutations detected? What data is there about the safety of any combinations? If there is, is that from this tumour type and would the data transfer to the specific disease my patients has? These myriad questions pose a major scientific challenge and yet patients are expecting decisions today. The Medical Innovation Bill (Saatchi) is being debated in the UK Parliament and seeks to remove constraints other than ‘the patient's best interests’ that could in any way limit the physician treating the patient. Such constraints include important issues including established practice, ethical permission for research, research protocols and research training. The Bill would enable a highly risky, unrestricted use of any licensed medication for any indication by a practicing doctor, with no requirement to publish outcomes. Of course the occasional success will be loudly trumpeted adding to hype and further undermining real evidence. Both news of breakthrough drugs and genomic print outs can distort clinical care in several ways. Explanation of existing care and evidenced based treatments options can be side-lined. Behaviours seeking ‘breakthrough’ medications can be extreme, including expensive, uninsured trans-continental travel, raising large sums sometimes by re-mortgaging the house, to support treatment seeking activities. The narrative that there is a personalised treatment for your disease if only your physician was smart enough or your health care system had invested enough is a disturbing and highly distressing perspective when faced with life threatening illness.

## A sober synergy of technology with patient care

As a research active physician, I attempt to find the right balance between two priorities. On the one hand, I actively work to identify stratifiers which will enable us to optimise the treatment for patients from the time of their first diagnosis. On the other in the clinic, I explain the disease and its treatment to my patients and help them to understand the reality of their situation, however difficult and limited that reality may be. Active engagement with the research agenda is compatible with prioritisation of the patient's own interests and indeed these can combine in a synergy which can be highly productive and satisfying. That synergy is best achieved when aims are aligned and research endpoints are inclusive of the primary desires of the patient — whether that be for improved survival, or simply the breath to be able to walk to the nearest shop. We are required to explain why the breakthrough on the media or the predictions of the genetic sequencing may not be realistic options and so manage expectations and indeed act as a firewall against hype. A research active clinician must therefore both act as an agent of change and development, but also a communicator of realism and a break on the hype. Thus personalised medicine that includes a sober appreciation of what genomics can achieve, together with continued focus on the individual as a person not just as a genome, will contribute to further improvements in health and healthcare.

## Related video

A video linked to this paper can be found at: http://www.healthcarevalues.ox.ac.uk/tim-maughan-promise-and-hype-personalised-medicine
